# The Basal Ganglia Downstream Control of Action – An Evolutionarily Conserved Strategy

**DOI:** 10.2174/1570159X21666230810141746

**Published:** 2023-08-10

**Authors:** Johanna Frost-Nylén, William Scott Thompson, Brita Robertson, Sten Grillner

**Affiliations:** 1 Department of Neuroscience, Karolinska Institute, Stockholm, Sweden

**Keywords:** Evolution, basal ganglia, dorsolateral striatum, substantia nigra pars reticulata, motor centers, corticostriatal, striatonigral

## Abstract

The motor areas of the cortex and the basal ganglia both contribute to determining which motor actions will be recruited at any moment in time, and their functions are intertwined. Here, we review the basal ganglia mechanisms underlying the selection of behavior of the downstream control of motor centers in the midbrain and brainstem and show that the basic organization of the forebrain motor system is evolutionarily conserved throughout vertebrate phylogeny. The output level of the basal ganglia (*e.g*. substantia nigra pars reticulata) has GABAergic neurons that are spontaneously active at rest and inhibit a number of specific motor centers, each of which can be relieved from inhibition if the inhibitory output neurons themselves become inhibited. The motor areas of the cortex act partially *via* the dorsolateral striatum (putamen), which has specific modules for the forelimb, hindlimb, trunk, *etc*. Each module operates in turn through the two types of striatal projection neurons that control the output modules of the basal ganglia and thereby the downstream motor centers. The mechanisms for lateral inhibition in the striatum are reviewed as well as other striatal mechanisms contributing to action selection. The motor cortex also exerts a direct excitatory action on specific motor centers. An overview is given of the basal ganglia control exerted on the different midbrain/brainstem motor centers, and the efference copy information fed back *via* the thalamus to the striatum and cortex, which is of importance for the planning of future movements.

## 1 INTRODUCTION

The basal ganglia are a crucial part of the forebrain together with the cortex, thalamus, and the dopamine system since they contribute importantly to allowing an animal to adapt its movements to dynamic behavioral requirements. The circuits in the midbrain, brainstem, and spinal cord can execute a variety of movements, from saccadic eye movements to locomotion and posture. What the forebrain adds is the ability to recruit these different motor circuits when it is behaviorally meaningful for the individual. The forebrain is critical for selection of the behavior needed in a specific situation, such as foraging or escape. The basal ganglia contribute importantly to the selection of behavior and motor learning but depend entirely on input from different cortical areas, the thalamus, and the modulatory input from the dopamine, 5-HT, and histamine systems. These systems work normally together, and it can be argued that the cortex is not understandable without the basal ganglia, and vice versa. Their mode of operation is intertwined. However, many mammals can perform a substantial part of their behavioral repertoire after lesions of the frontal lobes and even larger parts of the cerebral cortex [1, 2]. Below we will focus on the parts of the basal ganglia that are directly involved in the control of movements.

## 2 THE ORGANIZATION OF THE BASAL GANGLIA IS CONSERVED THROUGHOUT VERTEBRATE PHYLOGENY

The design of the basal ganglia is phylogenetically old. The basic circuit diagram has remained the same over the last 500 million years, as it is virtually identical in lamprey and mammals, and their evolutionary lines became separate at this time [3-9]. The lamprey represents the oldest group of now-living vertebrates, while primates are the most recent. The lamprey pallium (homologue of the mammalian cortex) was, until recently, considered to be mainly olfactory, but we now know that it has a motor area with projections to the striatum, midbrain, and brainstem/spinal cord as in mammals, in addition to visual and somatosensory areas [10-12]. Fig. (**1**) summarizes the overall connectivity within the basal ganglia in both the lamprey and mammals. The striatum, the input structure, receives input from the cortex/pallium *via* both pyramidal tract (PT) and intratelencephalic (IT) neurons, thalamus, and substantia nigra pars compacta (SNc).

95% of striatal neurons are GABAergic projection neurons (SPNs), half of which project directly to the output level of the basal ganglia, substantia nigra pars reticulata (SNr), and globus pallidus interna (GPi). These are referred to as direct pathway SPN neurons (dSPNs), while the other half of the SPNs project to globus pallidus externa (GPe), which in turn inhibits SNr. This pathway is referred to as the indirect pathway, and the SPNs are thus referred to as iSPNs. The dSPNs express substance P and dopamine receptors of the D1 type, and dopamine has an excitatory effect on dSPNs. The iSPNs express enkephalin and dopamine receptors of the D2 type, and dopamine instead inhibits iSPNs.

In both mammals and the lamprey, the dopamine neurons of the SNc project to the striatum, as well as the other basal ganglia subnuclei, and to downstream motor centers in the superior colliculus (tectum in lamprey) and the mesencephalic tegmentum (mesencephalic locomotor center (MLR) [13-16]. Individual dopamine axons may have one branch going to the striatum and another to the superior colliculus/tectum [17]. The SNc input originates from many structures, including the pedunculopontine (PPN) and the lateral tegmental nuclei, the cortex/pallium, the lateral habenula and the striosome compartment of the striatum [14].

The SPNs in both the lamprey and mammals have low excitability and tend to be silent at rest, as they express inward rectifiers potassium channels that are open under resting conditions, but close as they become depolarized by external input. They are thus designed (through evolution) to be difficult to activate. In contrast, the GABAergic neurons in the GPe, SNr, and GPi are all spontaneously active at rest. These spontaneously active output neurons target different midbrain/brainstem motor centers, providing tonic inhibition and thus preventing the recruitment of motor programs. When the dSPNs become activated, they inhibit the SNr/GPi neurons and thereby remove inhibition from the downstream motor centers that are free to become active.

The table in Fig. (**1**) documents the close similarity in terms of connectivity, transmitters, peptides, expression of ion channel subtypes and glutamatergic, GABAergic, and dopaminergic receptor subtypes, and the presence of all gross structures. The striking similarity between the lamprey and mammals implies that the basal ganglia have remained similar in design over hundreds of million years, and, presumably, that this basic design has served vertebrates well. The tonic inhibition of downstream motor centers has most likely been helpful in preventing unintended movement to be expressed.

## 3 THE MAMMALIAN BASAL GANGLIA

In primates, the capsula interna separates the dorsal striatum into two parts, the putamen, and the caudate nucleus. The caudal part of the caudate nucleus has similar properties to that of the caudal putamen, and the rostral part of the caudal nucleus is similar to the rostral putamen. The rodent basal ganglia are dominated by a large input compartment, the dorsal striatum, corresponding to the putamen/caudate nucleus in primates. The striatum is by far the largest part of the basal ganglia. One would estimate that the output nuclei of the basal ganglia SNr/GPi only have 1% of the total number of neurons relative to the striatum [18].

In rodents, the dorsal striatum can be subdivided broadly into three regions: 1) the dorsolateral striatum (DLS) or the somatomotor striatum, which is mainly concerned with the control of movement, and receives a major input from the motor areas in the frontal lobe, 2) the dorsomedial striatum (DMS), with input from cortical association areas and 3) the ventral striatum, with input from limbic areas. The latter two are involved in complex aspects of behavior such as cognition and emotions. In this context, we will limit our discussion to the DLS and its role in the control of movement.

## 4 THE DORSOLATERAL STRIATUM IS FURTHER SUBDIVIDED INTO MODULES

**Fig. (2):**
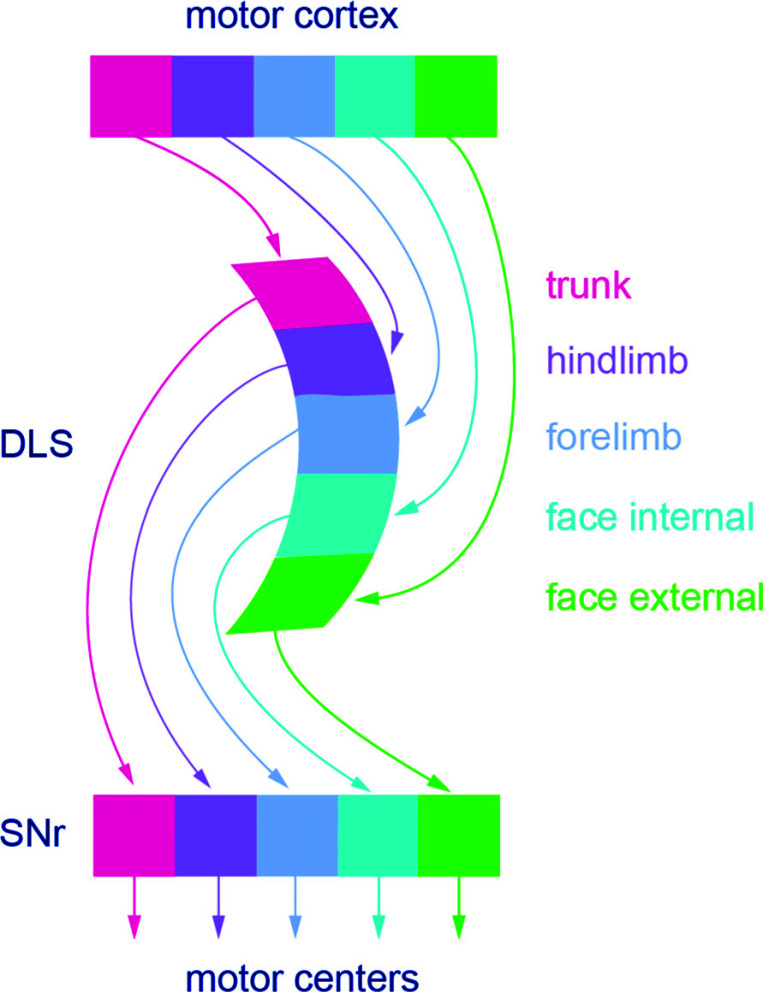
Modular connectivity from the motor cortex to the DLS and striatum. The modular nature of the connectivity from the different sections of the motor cortex to modules within the striatum and the connectivity *via* striatonigral dSPNs to modules within the SNr, which in turn each target different midbrain and brainstem motor centers. The indirect pathway *via* iSPNs (not illustrated) are subdivided into the same modules as the dSPNs and projects to discrete sets of GPe neurons (prototypic subsets) that in turn project to the same SNr modules as dSPNs.

The rodent DLS corresponds to the caudal parts of the putamen in primates. Recent findings [19] show that the DLS in the mouse can be further subdivided into discrete modules each with specific input from different parts of the cortical motor areas involved in the control of different discrete parts of the body such as the trunk, the hindlimb, and forelimbs, facial muscles including jaws, tongue and the interior of the mouth (Fig. **2**). More caudally in the DLS, eye movements are controlled. Fig. (**2**) illustrates the modularity from the cortex to DLS and further to compartments within the SNr. The latter represents the direct striatonigral pathway mediated by dSPNs. The indirect pathway mediated by iSPNs *via* the GPe to the SNr is also specific and follows the same modular rule [19]. In the mouse, the DLS is estimated to have around 300 000 neurons. The forelimb area is considered to roughly have 30 to 40 thousand neurons out of which roughly one-half projects directly to the output level (SNr) and the rest to the GPe. Optogenetic activation of dSPNS in the ventrolateral part of the striatum controlling the tongue can induce a licking response with short latency, most likely indicating that the effects are exerted on the downstream licking circuitry (CPG) in the brainstem *via* disinhibition due to the striatonigral inhibition of the SNr [20]. This means that activation of the striatonigral pathway in itself is sufficient to induce a motor behavior without concurrent effects from other structures such as the motor cortex.

## 5 INTERNAL CIRCUITS WITHIN THE STRIATUM

What internal processing takes place within the striatum? The striatum is essentially a GABAergic network without any intrinsic glutamatergic neurons and only a few cholinergic interneurons (ChINs). The excitation comes from the cortex and thalamus. To understand the operation of the striatal circuitry one needs to have information on the connectivity and the strength of the synaptic potentials between neurons. This requires recordings of pairs of presynaptic and postsynaptic neurons, a tedious but invaluable form of analysis [21, 22].

SPNs, in addition to their distally projecting axons, have local axonal ramifications around each neuron with a radius of approximately 150 μm. The local axons of SPNs target the very distal dendrites of other SPNs, where also the input synapses from the cortex and thalamus are located. Fig. (**3A**) shows the connectivity between dSPNs and iSPNs and how they interact and the proportion of neurons of each type that make synaptic contact at a given distance. Thus, dSPNs inhibit each other in 26% (red values) of the cases, and iSPNs inhibit each other in 36% of the cases. iSPNs also inhibit dSPNs in 28% of the cases, whereas the converse connection has a smaller value, only 6%.

**Fig. (3):**
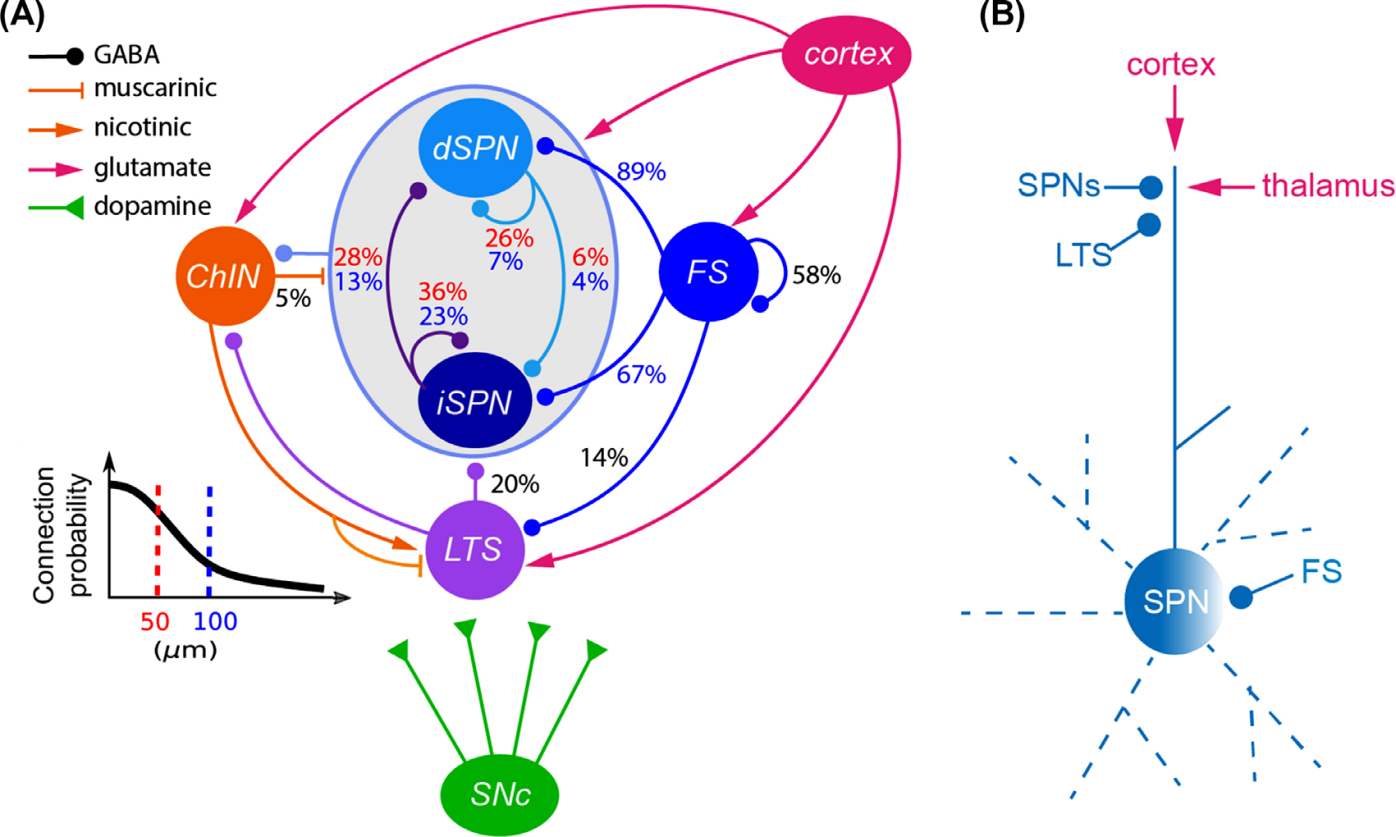
Striatal microcircuit. (**A**) Connectivity ratio between the two types of SPNs, the fast-spiking interneurons (FS), the low threshold spiking interneurons (LTS) and the cholinergic interneurons (ChINs). Connection probabilities within and between neuronal subtypes are shown by respective arrows; numbers in red correspond to connection probabilities for a somatic pair at a distance of 50 μm, while numbers in blue correspond to 100 μm. Input from the cortex is in red and SNc is in green. (**B**) An SPN shown graphically with input on the most distal dendrite from other SPNs, LTS and excitatory input from the cortex and thalamus. FS targets the soma area.

The different types of interneurons in the striatum represent each 1% or less than the total number of neurons in the DLS and will thus have less impact than the numerous SPNs. Fig. (**3A**) illustrates the fast-spiking interneurons (FS), the low threshold interneurons (LTS), and the ChINs that exert their effects *via* both nicotinic and muscarinic receptors. The FS have very tight connectivity: connection probabilities are reported as 89% to dSPNs and 67% to the iSPNs within a radius of 100 μm. In contrast to SPNs, they target the soma and proximal dendrites and will thus have an impact on spike initiation but not on the processing in the distal dendrites. The LTS contributes around 20% to SPNs in general, and as SPNs they target the distal dendrites of the SPNs (Fig. **3B**).

## 6 CORTICAL AND THALAMIC INPUTS TO SPNS

What matters for the integrated role of the striatum is the dynamic activity level (*i.e*. whether action potentials are generated) of the two subtypes of SPNs within each of the subpopulations of SPNs. This is determined both by the extrinsic excitatory input from the cortex and thalamus and by the intrinsic striatal inhibitory synaptic action onto SPNs. The dendritic trees of SPNs extend in all directions with the outer fine terminal branches representing 80% of the dendritic arbor. Both the excitatory input from the cortex and thalamus as well as the lateral GABAergic input from within the striatum make synapses primarily on these terminal dendrites (Fig. **3B**). The terminal dendritic branches are thus a major hub for synaptic integration and for inducing synaptic plasticity.

In SPNs, the excitatory synapses from the cortex and certain thalamic nuclei are almost exclusively on dendritic spines, whereas the input from the parafascicular thalamic nucleus (pf) is on both the dendritic shafts and on spines [23, 24]. The cortical input is *via* collaterals of brainstem-projecting PT neurons that create clusters of dense synapses in different areas of the striatum, and *via* the IT neurons that provide excitation both to PT neurons and directly to SPNs, with synapses distributed over a larger area [25, 26].

Under resting conditions, SPNs are silent since their membrane properties with inward rectifier potassium channels are hyperpolarizing. The SPNs are designed to have a high threshold for activation [27] in all vertebrates investigated [6]. It is thus important to consider which excitatory sources activate SPNs. The excitatory input from the cortex to DLS is most likely concerned with what can be referred to as a “proposal” to initiate a movement [28].

It must be recalled, however, that the input from the thalamus is almost as large as that of the cortex and it will affect the excitability of SPNs to an important degree. The input from pf is the most extensive, but several other thalamic nuclei also contribute. What type of information the thalamus relays to SPNs is known only to a limited extent. One part is the input fed back from the output neurons of the basal ganglia (SNr/GPi) in the form of an efference copy of downstream commands [29]. Another main input is from the different cerebellar output nuclei [30]. Furthermore, pf neurons also receive input from the superior colliculus, the PPN, the cortex, and the brainstem reticular formation [31, 32]. The pf neurons become activated by salient auditory stimuli, such as beeps and clicks, and by visual and somatosensory stimuli. The pf thus receives a mixed bag of information that can increase the excitability of SPNs of the DLS. How specific the different input channels of pf onto the DLS are, is at present unclear. The pf neurons have axons with rather large arbors that affect the main parts of the DLS [33]. Other thalamic nuclei have more clustered ramifications in the DLS, like those of the PT neurons. In addition to cortical and thalamic inputs, SPNs also receive input from the cholinergic neurons in the PPN [34, 35].

## 7 SYNAPTIC INTERACTION AT THE DISTAL DENDRITES OF SPNS

Focused cortical input to the distal dendrites can elicit plateau depolarization of SPNs dependent on activation of NMDA receptors, which can be transmitted to the soma and is important for the overall activity of SPNs [36]. The GABAergic inhibition from the surrounding SPNs and LTS (Fig. **3B**) can counteract the formation of these plateaus, perhaps terminating them earlier and thus helping to regulate the somatic response to distal input.

Even without plateaus, a marked GABAergic inhibition on the distal dendrites will increase the conductance of the dendritic membrane of the terminal dendrites and thereby shunt the EPSPs produced from the cortex and thalamus resulting in less depolarization at the level of the soma of the SPNs [37]. The processing in the distal dendrites is of critical importance since most synaptic processing takes place there (80%). The distal dendrites are thin (1-1.5 μm), which means that there will be a small intracellular volume in the subsynaptic region and that an intensive inhibitory GABAergic barrage may increase the chloride levels and thereby affect the inhibitory synaptic transmission. Whether the chloride level is maintained at the resting level depends entirely on the efficiency of the active transport of chloride *via* the KCC2 transporter [38, 39]. The level of the transporter is high in the dendrites. Whether the chloride equilibrium potential for chloride is maintained at the same level from the soma to the distal dendrites or if there is a gradient along the dendrites as has been suggested in other neurons is so far unclear. It is technically very difficult to record experimentally from the very thin dendrites of SPNs, and the analysis can so far only be explored through data-driven simulation [37, 40, 41].

Whenever a mouse is initiating a bout of locomotor activity or other types of movement there is a burst of activity in the dopamine neurons initiated by salient stimuli and perhaps the hope for some form of reward [42]. The impact of dopamine on dSPNs is excitatory *via* D1 receptors, whereas iSPNs will be inhibited *via* D2 receptors. This means that dSPNs will receive additional excitation, while the iSPNs will be depressed, therefore promoting action *via* the net difference in activity between the direct (go) and the indirect (no go) pathways.

The dopamine receptors are G-protein-coupled and therefore act with a somewhat longer delay than the action *via* the ionotropic glutamate receptors. Dopamine is therefore often referred to as a neuromodulator. The D1 receptors are coupled to G_olf,_ which activates adenylyl cyclase, which enhances the level of cyclic AMP (cAMP) that induces further downstream changes and activates DARRP 32, a “master switch” molecule that affects a number of downstream processes in the cell and different subtypes of ion channels [40, 43, 44]. The net effect of activation of D1 receptors in dSPNs is a depolarization partially depending on depression of potassium channels open at resting conditions. The D2 receptors are coupled to another G-protein, G_ialpha_ that inhibits the formation of cAMP, the net effect being a hyperpolarization of neurons with D2 receptors, such as iSPNs, and the cellular effects being opposite to those of D1 receptors. Dopamine neurons may not only release dopamine but also glutamate, which will promote the actions of D1 receptors on dSPNs [17, 45, 46].

Motor or procedural learning is another important task for the basal ganglia in which the dopamine system is playing a major role. Dopamine provides a feedback signal, communicating whether the motor task has been carried out in a desired way, or conversely in an unsatisfactory way. The activity level of the dopamine neurons in SNc is controlled by the striosomes in the striatum and other sources [47, 48]. Activation of D1 receptors in the dSPNs can promote synaptic plasticity and can if applied over a longer period, enhance the efficacy of synaptic transmission in a given cortico-dSPN synapse active during task performance, a form of long-term potentiation (LTP). The situation is different in iSPNs with D2 receptors, in which dopamine would promote long-term depression (LTD) of the synapses. However, adenosine can, *via* A2a receptors, promote LTP in iSPNs [49].

## 8 CAN LATERAL INHIBITION BETWEEN GROUPS OF SPNS CONTRIBUTE TO ACTION SELECTION?

If a population of SPNs becomes activated when a movement is being initiated, each spiking SPN will distribute inhibition within a radius of around 150 μm. Thus, around a center of active neurons, there will be lateral inhibition of surrounding SPNs, not being part of the active group of SPNs. Everything else being equal, these inactive neurons will become more difficult to activate from cortical, thalamic, or other excitatory sources. Lateral inhibition should therefore be able to counteract that nearby SPNs become activated, as long as the first group is active. The area of lateral inhibition is 150 μm in all directions around an active group of neurons. This inhibition would be able to have an impact on a surrounding striatal area corresponding to that of the fore- or the hindlimb module in the mouse, but very far from the entire DLS [37].

We must therefore conclude that lateral inhibition within the striatum may have a role in the control of the movement repertoire of a given DLS module, but not between the modules controlling different parts of the body. On the other hand, such an interaction may not be expected, since different types of movements can often be performed at the same time, most of us “can walk and chew gum at the same time”! In other situations, two movement alternatives are mutually exclusive - we cannot turn right and left at the same time. There is thus no room for interaction mediated through the SPNs between different parts of DLS. If long-range interactions do indeed occur in the striatum, it could only happen through interneurons such as the LTS that have long axonal arbors.

## 9 ACTION SELECTION WITHIN THE STRIATUM NOT REQUIRING LATERAL INHIBITION

As with the data from Foster *et al.* [19], referred to above, it seems that the different modules within the striatum each do their own thing, based on specific cortical, thalamic, and other inputs. It thus seems that the striatum consists of a mosaic of different modules that probably interact only to a limited extent. The dopamine neurons originating in the SNc, in contrast, have very extensive axonal arbors each covering a substantial part of the DLS. The effect of a dopamine burst may therefore extend over the entire DLS and not be specific to a given module within the DLS or be task specific.

The data of Foster *et al.* [19] implies that there is a “labeled line” from a given part of the motor cortex to subpopulations within the striatum and further to compartments within the SNr (for the dSPNs; Fig. **2**). During the initiation of a given movement, a set of striatal neurons becomes activated [50] dependent presumably on input from the cortical motor areas. Whether a cortical input to a subset of dSPNs will be efficient in recruiting them to firing action potentials may, however, be dependent on the concomitant thalamic input to the same SPNs, the local striatal interneurons, activity in surrounding SPNs, and the level of the dopamine and other modulators [49]. Fig. (**4**) shows a presumed motor command from the motor cortex that excites the SPNs, whether this will result in action potentials will depend entirely on the many different excitatory and inhibitory synaptic input that affects the SPNs at the same time.

If the dSPNs become active they will inhibit their targets in SNr and thereby, through disinhibition, promote activity in a given motor center, be it a saccadic eye movement, reaching, or locomotion. Counteracting activation of SPNs will instead result from activation of the local GABAergic interneurons and from the input of the arkypallidal neurons in the GPe that become active when a movement is being terminated. The arkypallidal neurons are therefore called stop cells.

If we assume that a group of PT neurons in the cortical motor areas (*e.g*. frontal eye field) that target neurons in the superior colliculus become activated, a saccade can be elicited. For this to occur, it will require that the tonic inhibition from SNr is removed through disinhibition. The PT neurons give off collaterals in the striatum and both PT and IT neurons target a population of SPNs that will become depolarized. If the dSPNs become activated, the striatonigral projection may cause the disinhibition required. However, processing within the striatum can also prevent this from occurring by lowering the excitability of the dSPNs under consideration such that they will not be recruited. This may be due, for instance, to external input from the thalamus (see section 7) or the PPN activating the GABAergic interneurons in the striatum, leading to a net inhibition of the dSPNs preventing them from initiating action potentials and thereby preventing the SNr disinhibition of motor centers to occur. In such a case, the saccade will not be elicited [51]. In this way, the intrastriatal processing may have served to select or rather prevent a given motor command from evoking a movement.

In summary, whether a given cortical command will be selected for action within the striatum will thus depend on the concurrent thalamic input to the specific dSPNs involved, the level of dopamine, and the activity within the pool of GABAergic interneurons, surrounding SPNs and the arkypallidal stop cells in GPe.

## 10 THE BASAL GANGLIA OUTPUT STAGE (SNR/GPI) AND THE CONTROL OF DOWNSTREAM MOTOR CENTERS

The output from the basal ganglia is to a large degree directed to downstream motor centers in the midbrain and brainstem in all vertebrates investigated, from lamprey to primates [6, 29, 52], a fact often overlooked by the research community that has focused on the projections back to the thalamus and further upstream. The output of the basal ganglia is mediated by the SNr and the entopeduncular nucleus (GPi). In rodents, the larger part (90%) is mediated by the SNr, but in primates, the GPi has a larger proportion of the efferent control.

In contrast to SPNs, the SNr neurons are tonically active under resting conditions. Different subpopulations of SNr neurons target separate midbrain/brainstem areas and thereby keep the different motor centers under continuous inhibition. There are a large number of motor centers that directly or indirectly, *via* command structures such as the MLR or the periaqueductal gray (PAG), control motor behavior [52, 53]. These include locomotion, eye movements, reaching, grasping, chewing, respiration, and escape/freezing [54]. The evolutionary significance of this inhibitory control of downstream motor centers is most likely that the forebrain can prevent a large number of motor centers from accidentally becoming active. As mentioned above, specific motor programs can be selectively called into action by being relieved from the inhibitory control, through disinhibition of the specific population of SNr neurons that inhibit the specific motor center concerned.

**Fig. (5):**
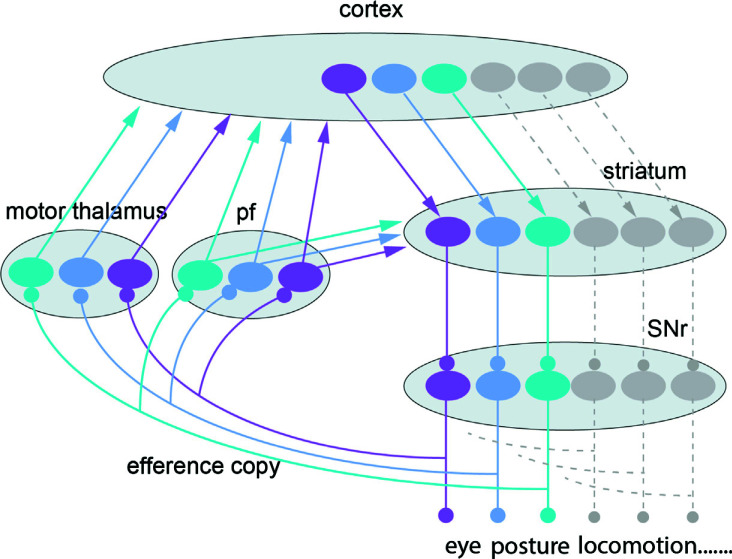
Schematic representation of the basal ganglia downstream control of motor structures in the midbrain-brainstem with specific efference copy information transmitted back *via* the thalamus to cortex and striatum. Subpopulations of neurons in the cortex project to subpopulations in the striatum that in turn inhibit discrete groups of neurons in the substantia nigra pars reticulata (SNr; [19]). Each circle indicates groups of neurons. Note that the upstream axonal branches to the motor thalamus and the parafascicular nucleus (pf) forward efference copies about the specific activity in the output channels. Only the “direct pathway” connectivity between the striatum and SNr is included in this scheme. McElvain *et al.*’s contribution is that SNr is subdivided into subpopulations with specific motor targets and that each conveys an efference copy to different parts of the motor thalamus or pf and further to the cortex and striatum.

McElvain *et al.* [29] have shown that the mouse SNr targets altogether 42 different groups of neurons in different parts of the midbrain, pontine, and medullary reticular formation, and sections of the superior and inferior colliculi. In a poetic sense, the SNr consists of many subpopulations akin to the many keys of a piano, each controlling a specific note (or for the SNr, a specific motor center). The neurons of each subgroup target a given downstream motor center but also emit an axonal branch that targets a given part of a thalamic nucleus, which in turn projects to either the striatum, cortex, or both (Fig. **5**). This type of information is often referred to as an efference copy. In this case, the efference copy carries information on the commands issued to the different motor centers before they have had time to be executed as actual movements. This should be indispensable information for further planning at the cortical or striatal level of how the next phase of the movement should be coordinated involving many components and parts of the body - consider for instance a cheetah hunting down prey.

The SNr is broadly subdivided into a lateral part that has input preferentially from the DLS, is engaged in the control of movement and consists primarily of parvalbumin (PV) positive neurons, and a medial part with input preferentially from the DMS, with PV negative neurons that target for instance neurons in the raphe nuclei. The membrane properties of SNr neurons differ, mostly being spontaneously active but with differences in firing rate, action potential shape, and various other electrophysiological parameters [29, 55]. PV-positive cells receive powerful input from the prototypic neurons of the GPe and the subthalamic nucleus (STN). The former targets the soma of SNr neurons and have markedly depressing synapses. They have a much weaker synaptic linkage to the medial, PV-negative SNr neurons. In contrast, striatonigral synapses are located on the distal dendrites of SNr neurons and are facilitating, and able to efficiently silence the SNr neurons. The STN input to SNr is glutamatergic and can increase the firing rate of SNr neurons [55] further inhibiting motor execution.

Lateral SNr neurons also receive excitatory monosynaptic input from the motor cortex [19, 56] and from neurons in the MLR area [57]. This means that the lateral part of SNr not only serves as the output of the basal ganglia but can be accessed by the cortex and MLR for direct control (halting movements).

## 11 THE GLOBUS PALLIDUS EXTERNA AND THE SUBTHALAMIC NUCLEUS AND THEIR ROLES IN THE INDIRECT PATHWAY

Our discussion above has mostly focused on the direct pathway with the striatonigral projections included in the “go”-pathway. As a movement is initiated from a part of the motor cortex, in most cases dSPNS and iSPNs within a certain striatal subpopulation become initially coactivated, but subsequently, the activity of the iSPNs diminishes markedly, while dSPNs can maintain their activity throughout the movement [58-60]. A concurrent dopamine burst will contribute to this asymmetry [42] by exciting dSPNs and inhibiting iSPNs. The net effect of the iSPN activation is an increase in SNr activity that will counteract the movement command.

The iSPNs project to the GPe and its prototypic GABAergic cells, which are spontaneously active at rest (20-30 Hz). These neurons will become inhibited by iSPNs when the indirect pathway is activated. This means that the spontaneously active prototypic cells become silenced or less active, and since they inhibit SNr, it means that SNr neurons will receive less inhibition and then increase their discharge rate.

**Fig. (6):**
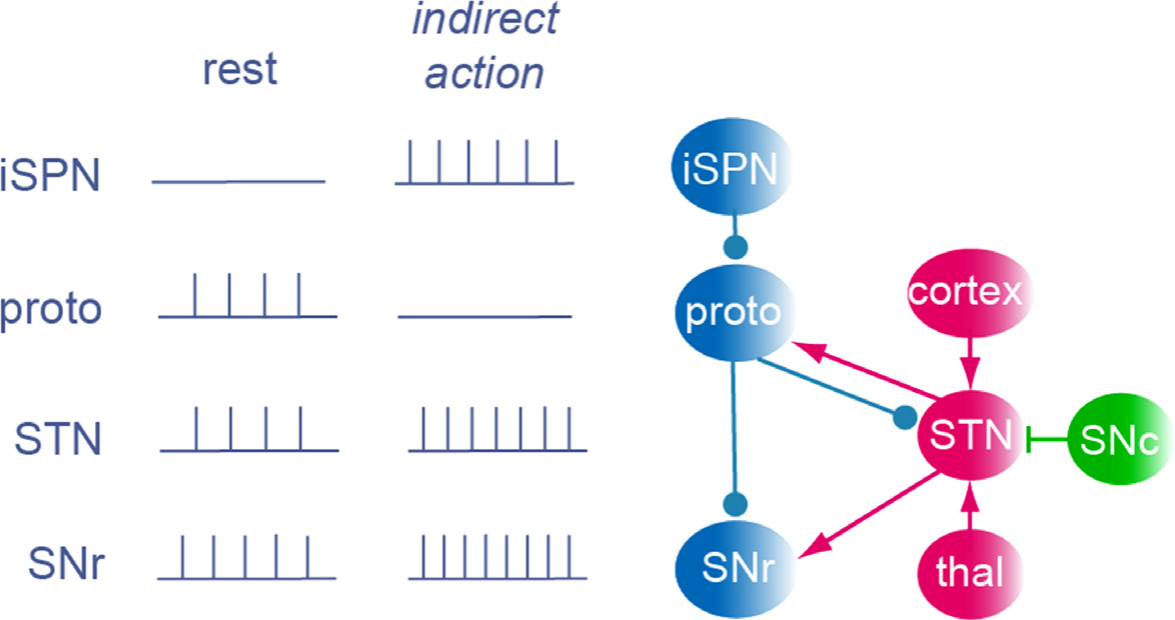
The connectivity of the indirect pathway and the subthalamic nucleus (STN), and activity under resting conditions and during activity in the different cell types. Under resting conditions, SPNs tend to be silent, but during bouts of behavior, they approach 40 Hz, while the neurons of the GPe (prototypic and arkypallidal), STN and SNr all have a resting level of discharge [6]. Proto, prototypic cells; thal, thalamus.

A further effect of the inhibition of the prototypic cells arises from the fact that they also inhibit the spontaneously active neurons in the STN [61]. These glutamatergic STN neurons also project to the SNr and can further enhance SNr activity (Fig. **6**). STN neurons themselves receive direct excitation from the cortex, a connection referred to as the hyperdirect pathway [62], a pathway that can stop movements through enhancing SNr activity. The STN also receives input from the thalamus and SNc (*via* D1/D5 receptors) [49]. The effects mediated *via* the STN input to SNr will be more unpredictable than those of the direct or indirect pathways, since they depend on the concurrent input from the cortex, thalamus, PPN and SNc. Moreover, enhanced activity in the STN directly excites the prototypic cells (Fig. **6**) would enhance their activity and cause a subsequent inhibition of the SNr (a counterintuitive effect).

The STN is subdivided into three parts, a motor, a cognitive and an emotional compartment. We limit here the discussion to the DLS-related part, and as noted above the STN projections to SNr is more prominent to the PV DLS-related part of SNr, similar to the lateralization of input from the GPe (prototypic cells).

The second main type of GPe neuron is the arkypallidal neuron, which expresses the transcription factor FoxP2. They are spontaneously active at a low rate and inhibited by the prototypic cells. When the indirect pathway is activated, they will thus become disinhibited. The arkypallidal neurons are GABAergic and project back to the striatum with a massive and extensive net of terminals. A direct projection from the motor cortex provides a very efficient activation of the arkypallidal neurons and in behavioral experiments, they become activated as the mouse terminates an action [63-68]. They are called “stop cells” and can very efficiently inhibit parts of the striatum. When the direct pathway is activated, these stop cells also receive inhibition from the axons of dSPNs, which agrees well with their perceived role in counteracting action [66].

The GPe also contains a separate set of neurons that receives input from dSPNs of the DMS. These neurons have an exclusive target, the pf nucleus of the thalamus, in contrast to the dSPNs of the DLS that target the SNr/GPi. The GPe-pf neurons project back to both the striatum and cortex - thereby bypassing the ordinary output route of the basal ganglia [69] and conveying information back to these structures.

## 12 POSSIBLE IMPLICATIONS OF THE CONVERGENCE OF THE INDIRECT AND DIRECT PATHWAYS IN SNR

Activity in the direct pathway is often combined with the initiation of behavior, while the indirect pathway is related to preventing movements (the no-go pathway) but they often become coactivated during a movement [58, 60, 70]. As we have noted above, the dSPNs and iSPNs from the same area of the striatum exert their effects on the same general area of SNr [19]. dSPNs act directly through the striatonigral projection and the iSPNs *via* the prototypic cells. This does not necessarily mean that they target the very same neurons in the SNr, but it allows for the possibility that the direct and indirect pathways act with opposite valence on the same individual SNr neuron. The activity of the SNr neurons during movement would then result from the relative balance between the two, the dSPN-induced inhibition and the iSPN-induced disinhibition exerted *via* the prototypic neurons. As we have noted above, initially during a movement both iSPNs and dSPNs may become activated but the iSPN activity then vanishes. The net result would be a promotion of SNr inhibition and therefore a disinhibition of the motor center being the target of the SNr neurons concerned. One can, however, not exclude the alternative explanation that dSPNs project to the SNr neurons that control the motor center to be activated, while the iSPNs indirectly affect SNr neurons affecting antagonist motor centers, a view often held. In this context, one should also consider the dopaminergic effects exerted on synaptic plasticity for instance the corticostriatal synapse onto dSPNs and iSPNs in terms of both short- and long-term synaptic depression and potentiation [70, 71].

## 13 DOES THE BASAL GANGLIA HAVE A PERMISSIVE ROLE IN THE CONTROL OF MOTION THROUGH THE DLS?

The ratio of neurons in the striatum and SNr/GPi is 100 to 1 [18] and if the DLS contains 300 000 neurons there should be around 30 00 SNr neurons. If a module like that for the striatal forelimb contains 40 000 neurons (estimated from [19]) there would be only approximately 400 neurons in SNr to provide control over the repertoire of different forelimb movements. This is of course a very rough estimate with a number of potential shortcomings. It nevertheless allows for the consideration that the basal ganglia downstream control of motor centers may rather be of the permissive type, lifting inhibition from a wider motor area. Further specifications may require concomitant input from the motor cortex to the motor circuits targeted, but also possible input from other structures such as the superior colliculus that can serve to further specify the movement [72], or the different motor centers contain intrinsic mechanisms for coordination. For instance, activating the MLR leads to the activation of the spinal locomotor CPGs that contain sufficient information to coordinate the entire step cycle [73].

## CONCLUSION

In this brief overview, we have noted that the basic design of the basal ganglia has remained very similar since the lamprey diverged from the evolutionary line leading to mammals some 500 million years ago, although the number of each type of neuron has increased manifold. In parallel, the richness of the behavioral repertoire has increased. The importance of lateral inhibition between SPNs probably contributes to selection only within a single DLS module such as that for the forelimb. It is also emphasized that gating of the motor commands from the cortex takes place in the striatum due to the multitude of concurrent inputs to SPNs from the thalamus, PPN, GABA interneurons, and the different modulators (dopamine, 5-HT, histamine, acetylcholine). If a cortical motor command leads to an activation of the striatonigral neurons this will result in the inhibition of the neurons in SNr and the disinhibition of a given downstream motor center. Subpopulations of SNr neurons each target one of the many motor centers in the midbrain-brainstem. This leads in turn to a disinhibition of the center and a release of motor action. Each SNr neuron also gives off a collateral to the thalamic nuclei and then further to the cortex and striatum. This information will be a copy of the downstream commands from SNr to the motor centers and therefore a form of efference copy of importance in the cortex/striatum for further motor planning.

## Figures and Tables

**Fig. (1):**
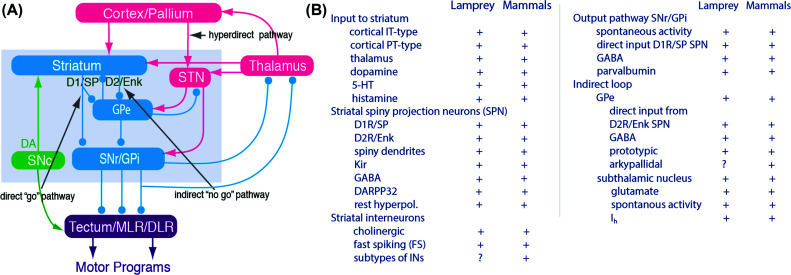
The basic organization of the lamprey and the mammalian basal ganglia is similar. (**A**) The general diagram of the basal ganglia applies to both lamprey and mammals. The striatum consists of GABAergic neurons, as do globus pallidus pars externa (GPe), globus pallidus pars interna (GPi) and substantia nigra pars reticulata (SNr). SNr and GPi represent the output level of the basal ganglia, and they project *via* different subpopulations of neurons to the tectum/superior colliculus, the mesencephalic (MLR), and diencephalic (DLR) locomotor regions, and other brainstem motor centers, as well as back to thalamus with efference copies of information sent to the brainstem. The direct striatal projection neurons (dSPNs) that target SNr/GPi express the dopamine D1 receptor (D1) and substance P (SP), while the iSPNs (indirect striatal projection neurons) express the dopamine D2 receptor (D2) and enkephalin (Enk). Also indicated is the dopamine input from the substantia nigra pars compacta (SNc; green) to the striatum and brainstem centers. Excitatory glutamatergic neurons are shown in pink and GABAergic structures in blue. (**B**) A table showing the key features of the basal ganglia organization that are found in mammals and lamprey.

**Fig. (4):**
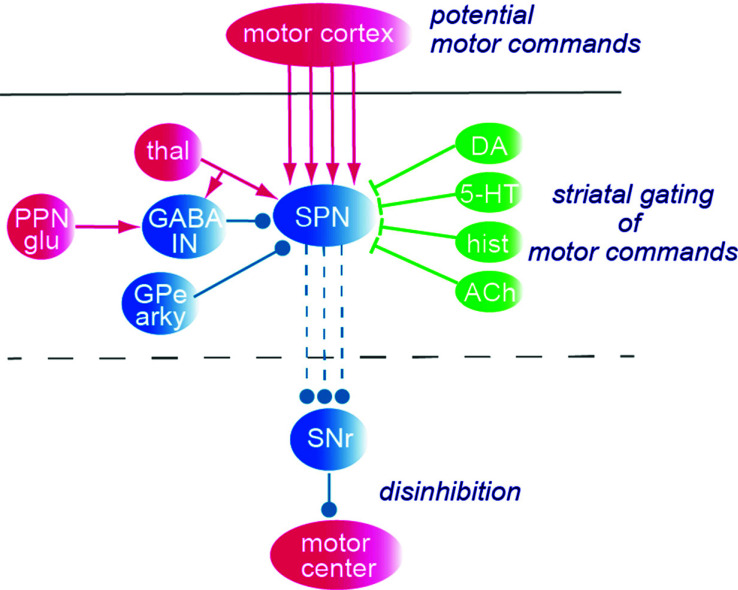
Whether a cortical motor command will be efficient in activating downstream SPNs will be determined by the activity in the many concurrent input channels in the striatum. ACh, acetylcholine; DA, dopamine; GABA IN, the different GABA interneurons in the striatum; GPe arky, the arkyplallidal neurons (stop neurons), hist, histamine; PPN glu, pedunculopontine nucleus, glutamatergic part; thal, thalamus; Red, glutamatergic neurons; blue, GABAergic neuron; green, the different modulators that target SPNs.

## LIST OF ABBREVIATIONS

ACh: Acetylcholine
CGP: Central Pattern Generator
ChINs: Cholinergic Interneurons
D1: Dopamine D1 Receptor
D2: Dopamine D2 Receptor
DA: Dopamine
DLR: Diencephalic Locomotor Region
DLS: Dorsolateral Striatum
DMS: Dorsomedial Striatum
dSPN: Direct Pathway Striatal Projection Neuron
Enk: Enkephalin
EPSP: Excitatory Postsynaptic Potential
FS: Fast-spiking Interneurons
GABA: Gamma-aminobuturic Acid
GPe: Globus Pallidus Externa
GPi: Globus Pallidus Interna
iSPN: Indirect Pathway Striatal Projection Neuron
IT: Intratelecephalic Tract Neuron
LTD: Long-term Depression
LTP: Long-term Potentiation
LTS: Low Threshold Interneurons
MLR: Mesencephalic Locomotor Region
NMDA: N-methyl-D-aspartate
PAG: Periaqueductal Gray
pf: Parafascicular Thalamic Nucleus
PPN: Pedunculopontine Nucleus
PT: Pyramidal Tract Neuron
PV: Parvalbumin
SNc: Substantia Nigra Pars Compacta
SNr: Substantia Nigra Pars Reticulata
SPN: Striatal Projection Neuron
STN: Subthalamic Nucleus
